# Is There a Correlation Between Reported Knee Pain and Fluid at the Distal Insertion of the Iliotibial Band in Runners?

**DOI:** 10.7759/cureus.28116

**Published:** 2022-08-17

**Authors:** Dusty Marie Narducci, Walter C Taylor, Daniel P Montero, Jennifer R Maynard, Christine Q Nguyen, Ryan Cudahy, George Pujalte

**Affiliations:** 1 Department of Family Medicine, University of South Florida Morsani College of Medicine, Tampa, USA; 2 Department of Family Medicine, Mayo Clinic, Jacksonville, USA; 3 Department of Orthopedic Surgery, Mayo Clinic, Jacksonville, USA

**Keywords:** runners, pain, iliotibial, fluid, distal

## Abstract

Objective

To determine whether there is a correlation between pain and the amount of fluid present at the distal insertion of the iliotibial band (ITB) in runners, as measured by USG.

Method

Our retrospective cross-sectional study evaluated 100 male and female runners prior to the start of a race. A valid and reliable questionnaire collected demographic, pain, and training data. If a runner reported knee pain, a numeric pain rating scale was used to record the degree of pain. Participants then underwent USG on both knees to determine the presence or absence of fluid at the distal insertion of the ITB.

Result

We found no statistically significant correlations of fluid measurements with pain score, running experience in years, or age. In addition, we found no other differences in fluid measurements between those with and without knee pain or between the sexes.

Conclusions

Our findings indicate that the presence or absence of fluid at the distal insertion of the ITB does not correlate with knee pain in runners, regardless of age, running experience, or sex.

## Introduction

Iliotibial band syndrome (ITBS) is one of the most common afflictions experienced by runners [1]. It is debatable what causes ITBS. Chronic inflammation of the iliotibial band (ITB) bursa has been a suggested cause for the condition [2], as well as compression of the connective tissue and fat under the ITB [3]. Repetitive extension and flexion of the knee causing friction between the lateral femoral condyle and the ITB is also felt to be a contributing cause [4]. The natural contracture and tightness of the ITB are presumed to correlate with the condition and its lateral symptoms [2]. Although a correlation between pain and the presence and amount of fluid in areas of musculotendinous pathology is commonly accepted [5], it is largely unknown whether the presence of such fluid at the distal insertion of the ITB is associated with pain. If a correlation exists between increased fluid at the distal ITB causing increased pain, it would be reasonable to assume that individuals with more fluid present at the distal ITB would have a higher pain score on the numeric rating scale (NRS).

Given that experienced runners may have better running and training techniques than novice runners [6], it could be assumed that experienced runners will have fewer ITBS symptoms and, perhaps, less fluid at the distal insertion of the ITB. Therefore, when knee pain is assessed, these individuals would also probably score lower on the NRS. As women are anatomically more predisposed to ITBS [7] due to wider hips resulting in different biomechanics than men [8], higher NRS scores on knee pain assessment would also be expected [9]. Older runners are more likely to have degeneration of the bursa located at the distal ITB [10]. Therefore, a decreased amount of fluid should consolidate at the distal insertion of the ITB. We hypothesized that pain measured using an NRS would have a positive correlation in different running populations as measured by the amount of fluid found by ultrasound at the distal insertion of the ITB.

## Materials and methods

For this retrospective cross-sectional study, 100 male and female participants were recruited during a weekend race event that featured a 5K, 10K, relay, half marathon, full marathon, and ultra-marathon (110 miles). Exclusion criteria were: 1) being under 18 years of age; 2) having previous knee surgery; 3) being a wheelchair user; 4) having received a knee injection within the previous three months, and 5) having already competed in one of the races during the race weekend. Informed consent was obtained from all participants, as stipulated by the Mayo Clinic Institutional Review Board (IRB number 16-010500). With a sample size of 100 participants, there was more than 80% power to detect a correlation coefficient of |0.3| or larger at the 5% significance level (two-sided).

Participants were asked to complete a survey asking about their age, BMI, presence of knee pain, an NRS score for knee pain (if present), previous diagnosis of any knee condition, and previous discontinuation of running due to pain. Participants were also asked about the average run duration, modification of training, running experience in years, weekly mileage, training pace, and the number of races in which they had participated.

**Figure 1:**
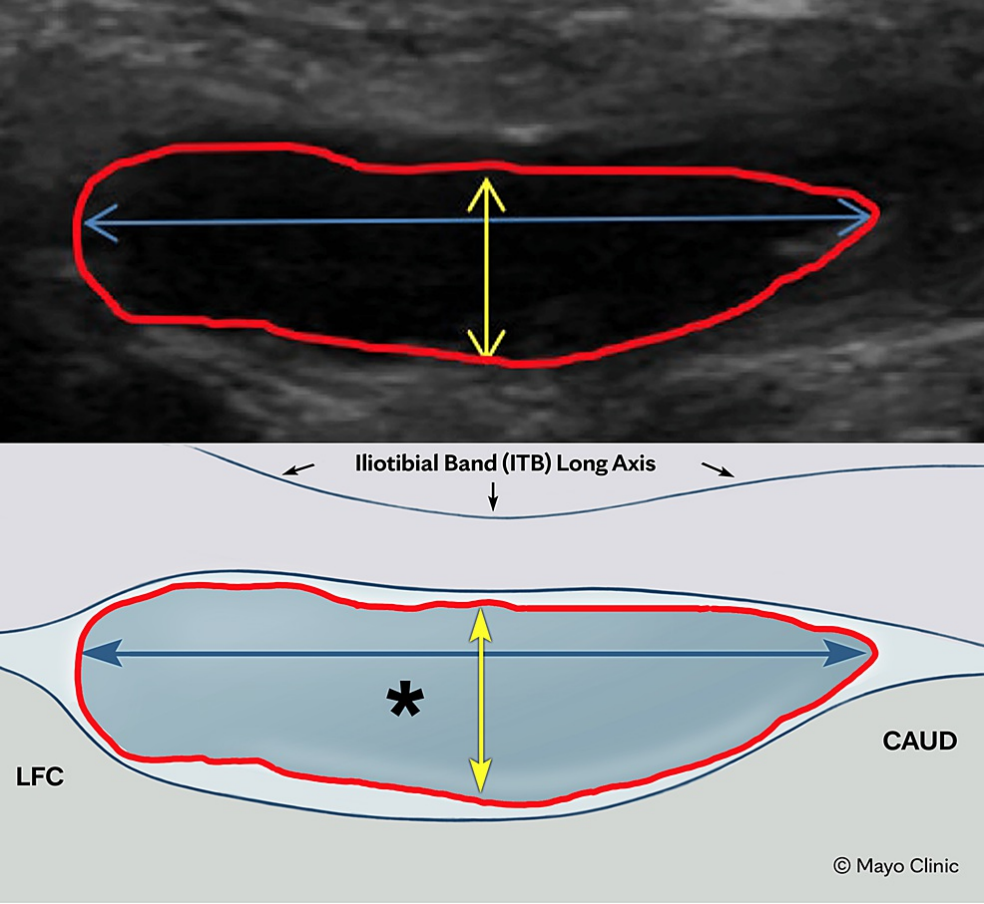
Ultrasound visualization of fluid measurement with representation of structures typically seen. Blue arrow (horizontal), long axis for area calculation by ultrasound machine (LAA). Yellow arrow (vertical), short axis for area calculation by ultrasound machine (SAA). Red line, circumferential calculation line by ultrasound machine. Short arrows, iliotibial band. Asterisk (*), ITB distal insertion fluid in subject. LFC: Lateral femoral condyle; CAUD: Caudal.

Fluid measurements were taken with the participant at 0° of extension and in the standing position. Sonographic measurements were obtained in the long-axis area of the left (LAA-L) and right (LAA-R) knees, the long-axis circumference of the left (LAC-L) and right (LAC-R) knees, and the short-axis area of the left (SAA-L) and right (SAA-R) knees (Figure 1).

This study had multiple hypotheses and aims: 1) Individuals with higher pain scores on the NRS would have more fluid present at the distal insertion of the ITB; 2) More experienced runners would have a less fluid and lower score on the NRS, validating to some extent, the belief that more experienced runners have better running and training techniques than novice runners; 3) Female runners would score higher on the NRS and have a greater amount of fluid present at the distal insertion of the ITB compared to male runners, given that female runners are anatomically predisposed to ITBS due to having wider hips; and 4) Older individuals would have less fluid present at the distal insertion of the ITB as a result of the degeneration of the bursa expected with age.

All analyses were performed using SAS, version 9.4 (SAS Institute Inc.). Survey responses and fluid measurements were descriptively summarized for all participants and separated according to sex. Spearman rank correlations (ρ) were used to evaluate the correlation of ITB fluid measurements with pain scores (Aim #1), years of running (Aim #2), and age (Aim #4), along with the correlation of pain scores with years of running (Aim #2). Wilcoxon rank-sum tests were used to compare pain scores and fluid measurements between males and females (Aim #3). As an additional analysis to supplement Aim #1, we compared fluid measurements between those with and without lateral knee pain present at the time of the survey using Wilcoxon rank-sum tests. P-values of 0.05 or less were considered statistically significant without adjustment for multiple testing.

## Results

Table 1 shows comparisons of fluid measurements between those with and without lateral knee pain at the time of the survey. Although not statistically significant, LAA-R and LAC-R were slightly higher in those with knee pain than those without (P=0.07 and P=0.08, respectively). There were no other differences in fluid measurements between those with and without knee pain (all P≥0.42).

**Table 1:** Comparison between those who did and did not report lateral knee pain at the time of the survey. *Wilcoxon rank-sum test †Fisher exact test

Characteristics	No knee pain (N=71)	Knee pain (N=28)	P-value
Age, years			0.341*
N	70	28	
Mean (SD)	46.1 (13.8)	49.3 (11.8)	
Median (range)	46.0 (18.0-73.0)	49.0 (24.0-72.0)	
Q1, Q3	38.0, 55.0	42.0, 57.0	
Sex, No. (%)			.50†
Male	26 (36.6)	8 (28.6)	
Female	45 (63.4)	20 (71.4)	
BMI			.71*
N	56	25	
Mean (SD)	24.3 (4.1)	24.5 (4.4)	
Median (range)	24.1 (9.8-36.8)	23.8 (19.2-36.1)	
Q1, Q3	21.6, 26.6	21.1, 26.6	
Long-axis area (left knee), cm^2^			.44*
N	71	28	
Mean (SD)	0.2 (0.2)	0.3 (0.3)	
Median (range)	0.2 (0.0-1.1)	0.2 (0.0-1.1)	
Q1, Q3	0.1, 0.3	0.1, 0.4	
Long-axis circumference (left knee), cm^2^			0.42*
N	71	28	
Mean (SD)	2.6 (1.8)	3.1 (2.2)	
Median (range)	2.5 (0.0-7.7)	2.8 (0.0-8.6)	
Q1, Q3	1.4, 3.8	1.7, 3.9	
Short-axis area (left knee), cm^2^			.54*
N	71	28	
Mean (SD)	0.2 (0.2)	0.2 (0.2)	
Median (range)	0.2 (0.0-1.2)	0.1 (0.0-0.8)	
Q1, Q3	0.1, 0.4	0.1, 0.3	
Short-axis circumference (left knee), cm^2^			.58*
N	71	28	
Mean (SD)	2.5 (1.5)	2.4 (1.6)	
Median (range)	2.7 (0.0-6.1)	2.2 (0.0-5.4)	
Q1, Q3	1.6, 3.5	1.6, 3.4	
Long-axis area (right knee), cm^2^			0.07*
N	71	28	
Mean (SD)	0.2 (0.2)	0.4 (0.3)	
Median	0.2 (0.0-0.8)	0.4 (0.0-1.2)	
Q1, Q3	0.1, 0.4	0.2, 0.5	
Long-axis circumference (right knee), cm^2^			0.08*
N	71	28	
Mean (SD)	2.8 (1.8)	3.6 (2.1)	
Median (range)	2.8 (0.0-6.8)	3.8 (0.0-7.5)	
Q1, Q3	1.6, 4.1	2.0, 4.8	
Short-axis area (right knee), cm^2^			.87*
N	71	28	
Mean (SD)	0.2 (0.2)	0.3 (0.3)	
Median (range)	0.2 (0.0-0.9)	0.2 (0.0-1.0)	
Q1, Q3	0.1, 0.4	0.1, 0.3	
Short-axis circumference (right knee), cm^2^			0.58*
N	71	28	
Mean (SD)	2.5 (1.5)	2.8 (2.0)	
Median (range)	2.5 (0.0-5.6)	2.7 (0.0-6.3)	
Q1, Q3	1.6, 3.8	1.6, 3.9	

Fluid measurements and survey responses are summarized by sex in Tables 2-3. When comparing male runners to female runners, there were no statistically significant differences in pain scores (median: male = 1, female = 2; P=0.58) or in any of the fluid measurements (LAA-L, P=0.74; LAC-L, P=0.40; SAA-L, P=0.24; SAC-L, P=0.18; LAA-R, P=0.42; LAC-R, P=0.44; SAA-R, P=0.70; SAC-R, P=0.81).

**Table 2:** Fluid measurements according to sex.

Characteristics	Female (N=66)	Male (N=35)	Total (N=101)
Age (years)			
N	65	35	100
Mean (SD)	45.8 (13.8)	48.7 (12.1)	46.8 (13.2)
Median	46	46	46
Q1, Q3	35.0, 55.0	42.0, 59.0	38.0, 56.0
Range	(18.0-73.0)	(19.0-72.0)	(18.0-73.0)
BMI			
N	54	29	83
Mean (SD)	23.9 (3.7)	25.0 (4.8)	24.3 (4.1)
Median	23.2	25.1	23.9
Q1, Q3	21.1, 25.1	23.1, 27.3	21.5, 26.6
Range	(19.2-36.8)	(9.8-36.1)	(9.8-36.8)
Long-axis area (left knee)			
N	66	35	101
Mean (SD)	0.3 (0.3)	0.3 (0.3)	0.3 (0.3)
Median	0.2	0.2	0.2
Q1, Q3	0.1, 0.3	0.0, 0.4	0.1, 0.3
Range	(0.0-1.1)	(0.0-1.1)	(0.0-1.1)
Long-axis circumference (left knee)			
N	66	35	101
Mean (SD)	2.9 (1.9)	2.6 (1.9)	2.8 (1.9)
Median	2.6	2.4	2.5
Q1, Q3	1.7, 3.8	1.4, 3.8	1.7, 3.8
Range	(0.0-8.6)	(0.0-6.5)	(0.0-8.6)
Short-axis area (left knee)			
N	66	35	101
Mean (SD)	0.2 (0.2)	0.2 (0.3)	0.2 (0.2)
Median	0.2	0.1	0.2
Q1, Q3	0.1, 0.3	0.0, 0.4	0.1, 0.3
Range	(0.0-0.8)	(0.0-1.2)	(0.0-1.2)
Short-axis circumference (left knee)			
N	66	35	101
Mean (SD)	2.6 (1.5)	2.2 (1.7)	2.5 (1.5)
Median	2.8	2.3	2.4
Q1, Q3	1.9, 3.5	1.0, 3.3	1.6, 3.4
Range	(0.0-5.9)	(0.0-6.1)	(0.0-6.1)
Long-axis area (right knee)			
N	66	35	101
Mean (SD)	0.3 (0.3)	0.3 (0.3)	0.3 (0.3)
Median	0.2	0.2	0.2
Q1, Q3	0.1, 0.4	0.1, 0.5	0.1, 0.4
Range	(0.0-1.2)	(0.0-1.1)	(0.0-1.2)
Long-axis circumference (right knee)			
N	66	35	101
Mean (SD)	3.2 (2.0)	2.8 (1.8)	3.1 (1.9)
Median	3	2.8	2.9
Q1, Q3	1.7, 4.5	1.5, 4.1	1.7, 4.4
Range	(0.0-7.5)	(0.0-6.6)	(0.0-7.5)
Short-axis area (right knee)			
N	66	35	101
Mean (SD)	0.2 (0.2)	0.2 (0.3)	0.2 (0.2)
Median	0.2	0.2	0.2
Q1, Q3	0.1, 0.4	0.1, 0.3	0.1, 0.3
Range	(0.0-1.0)	(0.0-0.9)	(0.0-1.0)
Short-axis circumference (right knee)			
N	66	35	101
Mean (SD)	2.6 (1.6)	2.5 (1.7)	2.6 (1.6)
Median	2.5	2.5	2.5
Q1, Q3	1.7, 3.8	1.4, 3.7	1.6, 3.8
Range	(0.0-6.3)	(0.0-6.2)	(0.0-6.3)

**Table 3:** Survey responses according to sex.

	Female (N=66)	Male (N=35)	Total (N=101)
1. Have you had lateral knee pain in the last five years?			
Missing	0	1	1
No	28 (42.4%)	14 (41.2%)	42 (42.0%)
Yes	38 (57.6%)	20 (58.8%)	58 (58.0%)
1b. If yes, which knee?			
Missing	28	15	43
Left	9 (23.7%)	8 (40.0%)	17 (29.3%)
Right	9 (23.7%)	3 (15.0%)	12 (20.7%)
Both	20 (52.6%)	9 (45.0%)	29 (50.0%)
2. Do you presently have pain in your lateral knee?			
Missing	1	1	2
No	45 (69.2%)	26 (76.5%)	71 (71.7%)
Yes	20 (30.8%)	8 (23.5%)	28 (28.3%)
2b. If yes, which knee?			
Missing	47	28	75
Left	5 (26.3%)	4 (57.1%)	9 (34.6%)
Right	12 (63.2%)	1 (14.3%)	13 (50.0%)
Both	2 (10.5%)	2 (28.6%)	4 (15.4%)
3. How would you rate your knee pain?			
Missing	2	1	3
0	27 (42.2%)	16 (47.1%)	43 (43.9%)
1	4 (6.3%)	3 (8.8%)	7 (7.1%)
2	6 (9.4%)	3 (8.8%)	9 (9.2%)
3	10 (15.6%)	6 (17.6%)	16 (16.3%)
4	7 (10.9%)	0 (0.0%)	7 (7.1%)
5	5 (7.8%)	3 (8.8%)	8 (8.2%)
6	5 (7.8%)	1 (2.9%)	6 (6.1%)
7	0 (0.0%)	1 (2.9%)	1 (1.0%)
10	0 (0.0%)	1 (2.9%)	1 (1.0%)
3. How would you rate your knee pain?			
N	64	34	98
Mean (SD)	2.0 (2.1)	1.9 (2.5)	2.0 (2.2)
Median	2	1	1
Q1, Q3	0.0, 4.0	0.0, 3.0	0.0, 3.0
Range	(0.0-6.0)	(0.0-10.0)	(0.0-10.0)
4. Were you given a diagnosis for this pain by a health care provider, either in the past five years or at present?			
Missing	1	0	1
No	49 (75.4%)	25 (71.4%)	74 (74.0%)
Yes	16 (24.6%)	10 (28.6%)	26 (26.0%)
4b. If yes, which diagnosis (choice=Iliotibial band syndrome)			
No	57 (86.4%)	29 (82.9%)	86 (85.1%)
Yes	9 (13.6%)	6 (17.1%)	15 (14.9%)
4b. If yes, which diagnosis (choice= Patellofemoral pain syndrome)			
No	65 (98.5%)	32 (91.4%)	97 (96.0%)
Yes	1 (1.5%)	3 (8.6%)	4 (4.0%)
4b. If yes, which diagnosis (choice=Meniscus injury)			
No	63 (95.5%)	35 (100.0%)	98 (97.0%)
Yes	3 (4.5%)	0 (0.0%)	3 (3.0%)
4b. If yes, which diagnosis (choice=Ligament injury)			
No	66 (100.0%)	35 (100.0%)	101 (100.0%)
4b. If yes, which diagnosis (choice=Other)			
No	63 (95.5%)	33 (94.3%)	96 (95.0%)
Yes	3 (4.5%)	2 (5.7%)	5 (5.0%)
4c. If other diagnosis, specify			
No	63 (95.5%)	33 (94.3%)	96 (95.0%)
Yes	3 (4.5%)	2 (5.7%)	5 (5.0%)
5. As a result of your knee pain, did you take time off from running?			
Missing	1	2	3
No	39 (60.0%)	21 (63.6%)	60 (61.2%)
Yes	26 (40.0%)	12 (36.4%)	38 (38.8%)
5b. If yes, for what duration?			
Missing	40	25	65
1 to 7 days	14 (53.8%)	4 (40.0%)	18 (50.0%)
8 to 30 days	2 (7.7%)	2 (20.0%)	4 (11.1%)
1 to 3 months	5 (19.2%)	1 (10.0%)	6 (16.7%)
4 to 5 months	1 (3.8%)	2 (20.0%)	3 (8.3%)
6 to 12 months	1 (3.8%)	1 (10.0%)	2 (5.6%)
More than 12 months	3 (11.5%)	0 (0.0%)	3 (8.3%)
5c. If more than 12 months, specify how long			
Missing	64	35	99
5 years	1 (50.0%)	0 (0.0%)	1 (50.0%)
Long years	1 (50.0%)	0 (0.0%)	1 (50.0%)
6. As a result of your knee pain, did you have to reduce your training?			
Missing	1	2	3
No	35 (53.8%)	17 (51.5%)	52 (53.1%)
Yes	30 (46.2%)	16 (48.5%)	46 (46.9%)
6b. If you continued to run despite your knee pain, what modifications did you make? (choice= none)			
No	63 (95.5%)	35 (100.0%)	98 (97.0%)
Yes	3 (4.5%)	0 (0.0%)	3 (3.0%)
6b. If you continued to run despite your knee pain, what modifications did you make? (choice= change in running technique)			
No	62 (93.9%)	32 (91.4%)	94 (93.1%)
Yes	4 (6.1%)	3 (8.6%)	7 (6.9%)
6b. If you continued to run despite your knee pain, what modifications did you make? (choice= change in running volume)			
No	54 (81.8%)	25 (71.4%)	79 (78.2%)
Yes	12 (18.2%)	10 (28.6%)	22 (21.8%)
6b. If you continued to run despite your knee pain, what modifications did you make? (choice= change in running intensity or pace)			
No	50 (75.8%)	28 (80.0%)	78 (77.2%)
Yes	16 (24.2%)	7 (20.0%)	23 (22.8%)
6b. If you continued to run despite your knee pain, what modifications did you make? (choice= change in shoe wear)			
No	58 (87.9%)	33 (94.3%)	91 (90.1%)
Yes	8 (12.1%)	2 (5.7%)	10 (9.9%)
6b. If you continued to run despite your knee pain, what modifications did you make? (choice= change in running surface or location)			
No	64 (97.0%)	33 (94.3%)	97 (96.0%)
Yes	2 (3.0%)	2 (5.7%)	4 (4.0%)
7. How many years have you been consistently running?			
Less than 1 year	3 (4.5%)	0 (0.0%)	3 (3.0%)
1 to 3 years	15 (22.7%)	7 (20.0%)	22 (21.8%)
4 to 5 years	14 (21.2%)	2 (5.7%)	16 (15.8%)
6 to 10 years	16 (24.2%)	12 (34.3%)	28 (27.7%)
More than 10 years	18 (27.3%)	14 (40.0%)	32 (31.7%)
7b. If more than 10 years, specify.			
Missing	54	24	78
11	1 (8.3%)	0 (0.0%)	1 (4.3%)
12	1 (8.3%)	0 (0.0%)	1 (4.3%)
15	1 (8.3%)	1 (9.1%)	2 (8.7%)
16	0 (0.0%)	1 (9.1%)	1 (4.3%)
18	0 (0.0%)	1 (9.1%)	1 (4.3%)
20	4 (33.3%)	1 (9.1%)	5 (21.7%)
21	0 (0.0%)	1 (9.1%)	1 (4.3%)
23	1 (8.3%)	1 (9.1%)	2 (8.7%)
25	1 (8.3%)	1 (9.1%)	2 (8.7%)
30	2 (16.7%)	0 (0.0%)	2 (8.7%)
32	0 (0.0%)	1 (9.1%)	1 (4.3%)
35	0 (0.0%)	1 (9.1%)	1 (4.3%)
38	1 (8.3%)	0 (0.0%)	1 (4.3%)
40	0 (0.0%)	2 (18.2%)	2 (8.7%)
8. What is your average weekly mileage?			
10 miles or less	20 (30.3%)	5 (14.3%)	25 (24.8%)
11 to 20 miles	29 (43.9%)	16 (45.7%)	45 (44.6%)
21 to 30 miles	15 (22.7%)	7 (20.0%)	22 (21.8%)
31 to 50 miles	2 (3.0%)	5 (14.3%)	7 (6.9%)
51 miles or more	0 (0.0%)	2 (5.7%)	2 (2.0%)
9. What is your average training pace (minutes/mile)?			
Missing	1	1	2
5	2 (3.1%)	0 (0.0%)	2 (2.0%)
7	2 (3.1%)	0 (0.0%)	2 (2.0%)
8	2 (3.1%)	6 (17.6%)	8 (8.1%)
9	8 (12.3%)	12 (35.3%)	20 (20.2%)
10	19 (29.2%)	7 (20.6%)	26 (26.3%)
11	12 (18.5%)	4 (11.8%)	16 (16.2%)
12	20 (30.8%)	5 (14.7%)	25 (25.3%)
9. What is your average training pace (minutes/mile)?			
N	65	34	99
Mean (SD)	10.4 (1.6)	9.7 (1.3)	10.1 (1.5)
Median	10	9	10
Q1, Q3	10.0, 12.0	9.0, 11.0	9.0, 12.0
Range	(5.0-12.0)	(8.0-12.0)	(5.0-12.0)
10. How many marathons have you run in your life?			
Missing	2	0	2
0	35 (54.7%)	11 (31.4%)	46 (46.5%)
1	6 (9.4%)	5 (14.3%)	11 (11.1%)
2 to 5	8 (12.5%)	3 (8.6%)	11 (11.1%)
6 to 10	10 (15.6%)	6 (17.1%)	16 (16.2%)
11 to 20	4 (6.3%)	7 (20.0%)	11 (11.1%)
21 or more	1 (1.6%)	3 (8.6%)	4 (4.0%)
11. How many half marathons have you run in your life?			
0	9 (13.6%)	3 (8.6%)	12 (11.9%)
1	7 (10.6%)	3 (8.6%)	10 (9.9%)
2 to 5	13 (19.7%)	6 (17.1%)	19 (18.8%)
6 to 10	13 (19.7%)	9 (25.7%)	22 (21.8%)
11 to 20	16 (24.2%)	10 (28.6%)	26 (25.7%)
21 or more	8 (12.1%)	4 (11.4%)	12 (11.9%)

Spearman correlations were used to evaluate correlations of each fluid measurement with pain score, years of running, and age. There were no statistically significant correlations of fluid measurements with pain score (all ρ≤0.11 and P≥0.19), years of running (all ρ≤0.08 and P≥0.42), or age (all ρ≤0.07 and P≥0.52).

## Discussion

ITBS is the most common presentation of ITB disease [10]. It is most commonly an overuse injury in runners, arising from the chronic compression of the ITB over a thin, richly innervated layer of fat, positioned between the ITB and the lateral femoral epicondyle [10]. Due to its diagnostic accuracy, wide availability, and affordability, USG is quickly becoming the first-line imaging modality in the assessment of ITBS [11]. It is largely unknown whether the presence of fluid in the area of concern in ITBS is a pathologic finding. Furthermore, it is unknown if the amount of fluid found in this region significantly correlates to the degree of symptoms compatible with ITBS. Therefore, this study aimed to investigate whether there was a statistically significant correlation between the presence and amount of fluid in the distal ITB region and ITBS symptoms, as well as the parameters that investigators believed may correlate with fluid. 

Our study demonstrated that fluid measurements in and around the distal ITB did not significantly correlate with pain score, sex, years of running, or runner age. There were no other differences in fluid measurements between those with and without knee pain. This information may help to characterize fluid in and around the distal ITB of ITBS sufferers as unrelated and perhaps help with a more thorough definition of sonographic findings that should be considered helpful in diagnosing ITBS. Peridistal ITB fluid is most likely incidental and should not factor into the diagnostic criteria for ITBS. Our study had certain limitations. Early pathologic findings (heterogeneity, increased echogenicity of the fat tissue between the band and femur) and more advanced stages (thickening and heterogeneity of the ITB itself) were not considered. However, we felt that the fluid in the distal ITB may not be as related to these changes as it would be to acute activities that inflame or damage the region [12]. Fluid measurements were limited as participants maintained a standing position with the knee in 0° extension only. It may have been more beneficial to measure fluid in a different position; however, the decision to measure distal ITB fluid in this manner was based on previous studies showing the highest likelihood of fluid detection in this position [13]. The risk of protocol deviation due to varying scanning techniques and different skill levels among the multiple study investigators may have also played a role in distorted measurements. Diagnosis may have also been less reliable due to compression of the transducer against the skin surface, possibly displacing the fluid. However, all investigators received the same well-designed protocol training before taking participants' measurements.

Additionally, the ultrasound machine determined the area of fluid being measured using the appropriate lines documented. The machine calculated the area and assumed it to correlate positively with the total fluid volume in the pocket, but this may not necessarily be true. The ultrasound machines used were identical models, tested prior to use, of great quality, and similar to the models used widely by practitioners in the field. Future research should focus on specifying anatomic parameters that may change fluid presence in other regions of the knee and fluid presence associated with runners' knee pain that may be detectable using sonography. Contributors to larger knee circumference (cartilage thickness, synovial layer thickness, synovial viscosity) could be individually examined in future studies.

## Conclusions

There are no statistically significant correlations of fluid measurements with pain score, sex, years of running, or age. There were no other differences in fluid measurements between those with and without knee pain. Therefore, the presence or absence of fluid at the distal insertion of the ITB is likely not a predictor of knee pain in runners, regardless of age, running experience, or sex. Bogginess or swelling noted clinically at this location, especially in runners, in the context of complaints compatible with ITBS, may be irrelevant in this population.
